# Digital PCR provides sensitive and absolute calibration for high throughput sequencing

**DOI:** 10.1186/1471-2164-10-116

**Published:** 2009-03-19

**Authors:** Richard A White, Paul C Blainey, H Christina Fan, Stephen R Quake

**Affiliations:** 1 Department of Bioengineering at Stanford University and Howard Hughes Medical Institute, Stanford, California 94305, USA

## Abstract

**Background:**

Next-generation DNA sequencing on the 454, Solexa, and SOLiD platforms requires absolute calibration of the number of molecules to be sequenced. This requirement has two unfavorable consequences. First, large amounts of sample-typically micrograms-are needed for library preparation, thereby limiting the scope of samples which can be sequenced. For many applications, including metagenomics and the sequencing of ancient, forensic, and clinical samples, the quantity of input DNA can be critically limiting. Second, each library requires a titration sequencing run, thereby increasing the cost and lowering the throughput of sequencing.

**Results:**

We demonstrate the use of digital PCR to accurately quantify 454 and Solexa sequencing libraries, enabling the preparation of sequencing libraries from nanogram quantities of input material while eliminating costly and time-consuming titration runs of the sequencer. We successfully sequenced low-nanogram scale bacterial and mammalian DNA samples on the 454 FLX and Solexa DNA sequencing platforms. This study is the first to definitively demonstrate the successful sequencing of picogram quantities of input DNA on the 454 platform, reducing the sample requirement more than 1000-fold without pre-amplification and the associated bias and reduction in library depth.

**Conclusion:**

The digital PCR assay allows absolute quantification of sequencing libraries, eliminates uncertainties associated with the construction and application of standard curves to PCR-based quantification, and with a coefficient of variation close to 10%, is sufficiently precise to enable direct sequencing without titration runs.

## Background

A new generation of sequencing technologies based on sequencing by synthesis and sequencing by ligation are revolutionizing biology, biotechnology, and medicine [1,2]. A key advance facilitating higher throughput and lower costs for several of these platforms was migration from the clone-based sample preparation used in Sanger sequencing to the massively parallel clonal PCR amplification of sample molecules on beads (Roche 454 and ABI Solid) or on a surface (Solexa) [3,4]. The workflow for these new sequencing technologies proceeds as follows: library creation, library quantification, massively parallel clonal PCR amplification of library molecules, and sequencing. During library creation, adaptor sequences are appended to both ends of the DNA molecules in a sample. The presence of these adaptors enables the amplification of random-sequence inserts by parallel PCR amplification of millions of individual DNA molecules. On the Roche/454 and ABI/SOLiD platforms, emulsion PCR is used to amplify a single DNA molecule to millions of copies of the same sequence all attached to a single polymer bead. On the Illumina/Solexa platform, library molecules are captured by surface-tethered probes complementary to the adaptor sequences and are amplified by bridge PCR to convert a single DNA molecule into a surface-bound cluster with many copies of the same sequence.

Accurate quantification of the number of library molecules is a critical factor affecting next-generation sequencing performance. Underestimation of library concentration results in multiple library molecules associating with the same bead within an emulsion microdroplet or overlapping images of DNA clusters after bridge PCR. The consequences are mixed signals or un-resolvable clusters, which reduce the number of high quality reads. Overestimation of library concentration results in fewer DNA-bearing beads after emulsion PCR or sparse clustering in bridge PCR, in which case the full capacity of the sequencer cannot be realized. Accurate quantification of the sequencing library is essential to achieve high yield and high quality sequencing. Inaccuracy in quantification is addressed by the manufacturers through 'titration' runs of the sequencer, which are used to empirically divine the concentration of productive DNA fragments in the sequencing library. The accuracy of digital PCR and its ability to count only amplifiable molecules obviate the need for expensive and time-consuming titration sequencing runs.

The manufacturers' protocols call for quantification of sequencing libraries by mass using capillary gel electrophoresis or UV spectrophotometry. The measured mass is converted to a molecule count using information about the fragment length distribution. These quantification techniques consume billions of library molecules at minimum (ng of DNA, see Table 1), about a thousand times more library molecules than are needed for sequencing. This is the reason that manufacturers require one to ten trillion DNA fragments (1 – 5 micrograms of DNA) as input for library preparation: to ensure that enough library molecules are obtained to allow for quantification. The requirement for micrograms of input DNA limits the pool of samples that can be analyzed with next generation sequencing technologies, since microgram quantities of genetic material is not available for many sample types, including some ancient, forensic, environmental, and clinical samples [5]. In a subset of cases, it is possible to amplify the input materials with PCR or multiple displacement amplification (MDA), but amplification introduces bias, distorting the representation of sequences [6]. To extend the application of next-generation sequencing and reduce the cost of sequencing, a technique for accurate quantification of dilute sequencing libraries is needed.

**Table 1:** Comparison of current sequencing library quantification methods

**Method:**	**Nanodrop**	**Capillary GE**	**Ribogreen**	**Real-time PCR**	**UT-qPCR**	**UT-digital qPCR**
**Detection Chemistry:**	UV absorption	Intercalating fluorophore	Intercalating fluorophore	Syber Green I Intercalating fluorophore	Hydrolysis probe (Taqman)	Hydrolysis probe (Taqman)
**Companies:**	Thermo Scientific	Aligent, Bio-Rad	Invitrogen	Many	Many	Fluidigm
LOQ:*	2 ng** (7.2 billion copies)	25 ng (91 billion copies)	1 ng (3.6 billion copies)	(0.3 fg) 1000 copies	(0.03 fg) 100 copies	(0.03 fg) 100 copies
**Quantification Modality:**	Mass/absolute	Mass/relative	Mass/relative	Mass/relative	Molecules/relative	Molecules/absolute
**Quantification Standard:**	No standard necessary	Required – calibrated by mass	Required – calibrated by mass	Required – calibrated by mass	Required – calibrated by mass	No standard necessary
**Reference:**	nanodrop.com	Ricicova, M. et al (2003)	Jones, Lj et al. (1998)	Simpson (2000); Meyer (2008)	Zhang (2003); This work	Kalinina (1997); This work

* Limit of quantification for ssDNA 500-mer;

** Manufacturer does not specify this value as LOD or LOQ

We developed a digital PCR-based method for highly accurate absolute quantification of sequencing libraries that consumes subfemptogram amounts of library material. Digital PCR is a technique where a limiting dilution of the sample is made across a large number of separate PCR reactions such that most of the reactions have no template molecules and give a negative amplification result. In counting the number of positive PCR reactions at the reaction endpoint, one can count the individual template molecules present in the original sample one-by-one [7]. The term 'digital PCR' was coined in 1999 [8]. A major advantage of digital PCR is that the quantification is independent of variations in the amplification efficiency – successful amplifications are counted as one molecule, independent of the actual amount of product. PCR-based techniques have the additional advantage of only counting molecules that can be amplified, *e.g. *that are relevant to the massively parallel PCR step in the sequencing workflow. Because digital PCR has single molecule sensitivity, only a few hundred library molecules are required for accurate quantification. Elimination of the quantification bottleneck reduces the sample input requirement from micrograms to nanograms or less, opening the way for minute and/or precious samples onto the next-generation sequencing platforms without the distorting effects of pre-amplification. Here we demonstrate the utility of digital PCR to directly prepare trace (<1 microgram) DNA samples for bulk sequencing on the 454/Roche and Solexa/Illumina sequencing platforms.

## Results

### Universal Template Taqman PCR assay design

**Figure 1:**
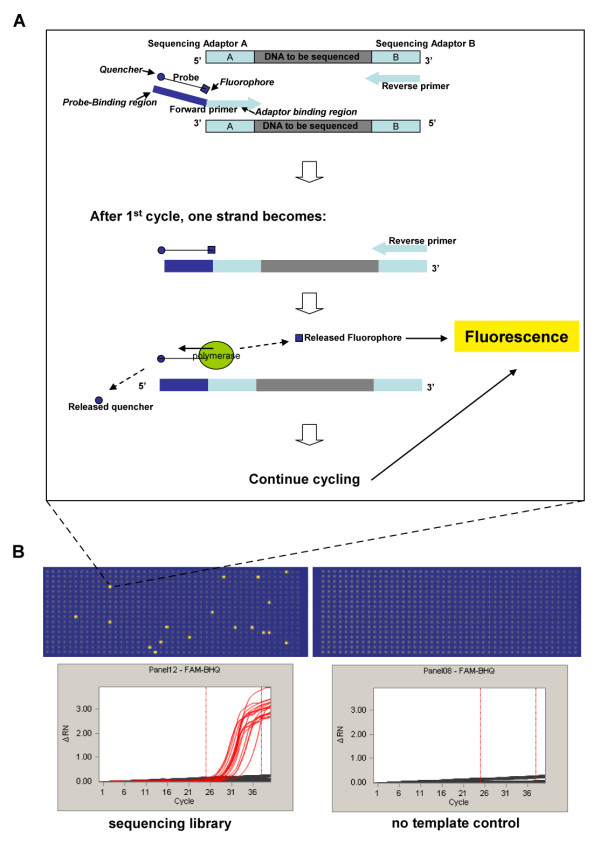
**A Schematic of the universal template (UT) PCR assay**. The forward primer (as drawn) includes a short sequence complementary to the 8 bp dual-labeled locked nucleic acid probe on its 5' end, followed by the sequence of one of the adaptors ligated to the library molecules on its 3' end. The reverse primer (as drawn) is complementary to the sequence of the other adaptor. As the polymerase encounters the probe during strand extension, its 5' to 3' exonuclease activity cleaves the probe, releasing the fluorophore from its quencher, thus producing fluorescent signal by dequenching. **B **The assay is performed on a commercial microfluidic digital PCR chip. At the end of the PCR, compartments that contain amplifiable DNA molecules with sequencing adaptors properly appended give positive signal, while compartments that do not remain dark. The count of positive compartments corresponds to the number of productive library molecules in the volume loaded onto the microfluidic chip, thereby allowing measurement of the concentration of amplifiable library molecules.

TaqMan PCR has the advantage of yielding a fluorescence signal proportional to the number of molecules that have been amplified, rather than the total mass of dsDNA in the sample [9]. This method works by the addition of a double-labeled oligonucleotide probe in a PCR reaction powered by a polymerase with 5' to 3' exonuclease activity. The probe must be complementary to one of the two product strands such that the extending polymerase will encounter the probe and cleave it, separating the probe's two labels and activating the probe's fluorescence through its exonuclease activity. Conventional TaqMan detection chemistry requires that the probe is complementary to the region within the amplified portion of the template between the two amplification primers. This strategy is not applicable to the sequencing libraries, which have inserts of unknown or random sequence between short adaptor sequences. To overcome the challenge of probe design for templates of random sequence, we adapted the universal template (UT) approach where a probe-binding sequence is appended to one of the PCR primers [10] (Figure 1A). One amplification primer includes a short sequence complementary to the probe on its 5' end, followed by a sequence complementary to one of the sequencing adaptors ligated to the library molecules. The second amplification primer in the UT scheme is complementary to the sequence of the second sequencing adaptor. To decrease reaction times, we replaced the published 20 bp UT probe-binding region with an 8 bp sequence target for a probe containing a locked nucleic acid nucleotide as applied in Roche's UPL (Universal Probe Library) probes (see methods for details). The shorter amplicon-probe interaction length allows the reduction of PCR run times from 2.5 hours to less than 50 minutes. In practice, we often use the UT-quantitative PCR assay in the real-time mode (with a calibration standard) to range the library concentration so that an appropriate dilution can be made for absolute quantification by UT-digital PCR.

### UT-digital PCR assay for sequencing library quantification

**Figure 2:**
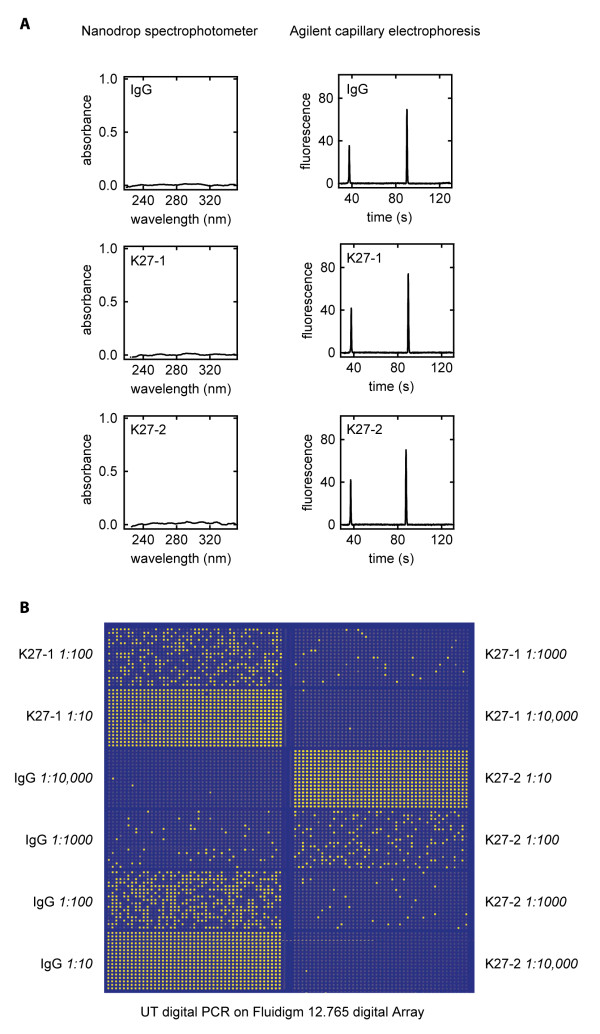
**Detection of three trace chromatin immunoprecipitate 454 single-stranded DNA libraries prepared from ~1.2 ng input mouse chromatin by digital PCR**. **A **No signal from libraries on the NanoDrop spectrophotometer or the Agilent Bioanalyzer capillary electrophoresis unit. The two signals appearing in the electropherograms are molecular weight markers of 15 bp and 1500 bp. **B **Detection of library molecules by digital PCR. False-color image of 12.765 digital array at assay endpoint. Each grid point corresponds to a nanoliter-scale PCR reaction, with yellow squares revealing amplification due to the presence of at least one sequencing library template molecule. The panels show dilution series (indicated) of samples analyzed in part A, allowing accurate absolute quantification of the samples by UT digital PCR.

The UT-digital assay is performed on a commercial microfluidic digital PCR chip from Fluidigm. The microfluidic system automates partitioning of 12 independent samples into 9,180 nanoliter PCR reactions and runs all those reactions simultaneously within the microfluidic chip. At the end of the PCR, compartments that contained DNA molecules with sequencing adaptors properly appended give positive signal, while compartments that did not remain dark. Figure 1B shows the results of a sample microfluidic digital PCR assay where 18 sequencing library molecules were counted on one panel (765 PCR reactions) of the chip and zero molecules were counted among the 765 PCR reactions on a negative control panel. The count of positive compartments corresponds to the count of library molecules in the volume loaded onto the microfluidic chip, allowing the concentration of library molecules to be determined. The sensitivity of the UT-digital PCR assay is demonstrated in Figure 2, where two trace libraries, undetectable at stock concentration by UV spectrophotometry or capillary gel electrophoresis, were accurately quantified at dilutions of 1:100 and 1:1000 by UT-digital PCR. We detected no digital PCR counts in a mock (no sample) 454 library preparation [see Additional file 1], indicating that the digital assay is a background-free measurement. As such, the observed digital counts arise from real sequencing library molecules, and accurately represent the number of amplifiable library molecules in the sample.

### UT-Digital PCR assay enables direct bulk sequencing of trace samples on 454

**Figure 3:**
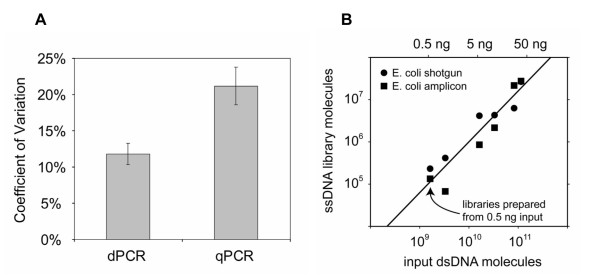
**Library quantification results**. **A**. Reproducibility of UT-PCR assays. Twelve 454 libraries were assayed with six to eight replicates by both UT-digital PCR and UT-quantitative PCR. UT-quantitative PCR was calibrated using a library quantified by digital PCR. The CV for dPCR is significantly lower than that for qPCR. **B**. Accurate digital PCR quantification of 454 libraries from trace quantities of input *E. coli *genomic or amplicon DNA. *E. coli *shotgun and amplicon DNA were first quantified by mass-based methods and the indicated amounts (0.5 to 35 ng) used for library preparation. The input quantity and yield are correlated with R^2 ^= 0.88. The library yield was assessed based on replicate UT-digital PCR quantification. Useful numbers of library molecules were recovered in all cases.

Digital PCR gives an absolute, calibration-free measurement of the concentration of amplifiable library molecules. To demonstrate the utility of digital PCR in preparing sequencing libraries from small amounts of starting material, twelve libraries were created from starting amounts of *E. coli *DNA ranging from 35 ng to as low as 500 pg. Six of the libraries were constructed with *E. coli *genomic DNA and six were prepared from the same quantities of an *E. coli *16S rRNA amplification product (of 466 bp), all according to the 454 shotgun protocol using molecular barcodes, "MIDs" (or Multiplex IDentifiers, which allow multiple samples to be mixed and sequenced as a pool). The resulting DNA libraries, undetectable by UV spectrophotometry or capillary gel electrophoresis, were quantified by UT-digital PCR. Useful numbers of library molecules were recovered from all twelve library preparations. The quantity of input DNA and the library yield for each sample is listed in Table 2 and shown in Figure 3A.

**Table 2:** Trace microbial/human 454 FLX library construction

***Sample ID***	***Input (ng)***	***Mean library fragment size (bp)***	***Input (total molecules by mass)***	***ssDNA library (total molecules by UT-dPCR)***	**dPCR replicate CV**	***Library prep recovery %***	***Library type***	***Organism***
***TS-1***	35 ng	500	1.19 × 10^11^	2.61 × 10^7^	7.07%	0.022%	Shotgun	*E. coli*
***TS-2***	25 ng	500	8.52 × 10^10^	6.15 × 10^6^	8.24%	0.007%	Shotgun	*E. coli*
***TS-3***	10 ng	500	3.41 × 10^10^	4.20 × 10^6^	7.71%	0.013%	Shotgun	*E. coli*
***TS-4***	5 ng	500	1.70 × 10^10^	4.08 × 10^6^	5.65%	0.024%	Shotgun	*E. coli*
***TS-5***	1 ng	500	3.41 × 10^9^	4.08 × 10^5^	16.84%	0.012%	Shotgun	*E. coli*
***TS-6***	500 pg	500	1.70 × 10^9^	2.31 × 10^5^	5.58%	0.014%	Shotgun	*E. coli*
***TS-7***	35 ng	466	1.27 × 10^11^	2.67 × 10^7^	8.55%	0.023%	Amplicon	*E. coli*
***TS-8***	25 ng	466	9.10 × 10^10^	2.11 × 10^7^	8.50%	0.025%	Amplicon	*E. coli*
***TS-9***	10 ng	466	3.64 × 10^10^	2.15 × 10^6^	4.90%	0.006%	Amplicon	*E. coli*
***TS-10***	5 ng	466	1.82 × 10^10^	8.49 × 10^5^	7.10%	0.005%	Amplicon	*E. coli*
***TS-11***	1 ng	466	3.64 × 10^9^	8.67 × 10^4^	21.90%	0.002%	Amplicon	*E. coli*
***TS-12***	500 pg	466	1.82 × 10^9^	1.31 × 10^5^	7.60%	0.007%	Amplicon	*E. coli*
***IgG***	~1.2 ng^a^	180	1.18 × 10^11^	3.24 × 10^6^	17.61%	0.003%	Shotgun	*M. musclus*
***K27-1***	~1.2 ng^a^	180	1.01 × 10^11^	1.87 × 10^6^	9.40%	0.002%	Shotgun	*M. musclus*
***K27-2***	~1.2 ng^a^	180	8.41 × 10^10^	1.49 × 10^6^	5.65%	0.002%	Shotgun	*M. musclus*
***Ace***	723 ng	550	2.26 × 10^12^	3.63 × 10^8^	6.10%	0.016%	Shotgun	*A. longum*
***pX***	4.9 ng^b^	180	9.26 × 10^10^	9.06 × 10^6^	4.60%	0.010%	Shotgun	*H. sapien*

^a ^DNA samples obtained from chromatin immunoprecipitation (ChIP) experiments. 2000 mouse cells were used for each experiment. The amount of DNA used for 454 library preparation is estimated by assuming 6 pg per cell and ~10% of the genome captured by a typical ChIP experiment. The input for library prep was undetectable by Nanodrop and Agilent Bioanalyzer.

^b ^Quantified by digital PCR using human specific primers at a unique locus, assuming 6.6 pg per cell equivalent. The input for library preparation was undetectable by Nanodrop and Agilent Bioanalyzer.

To assess the reproducibility of the UT-digital PCR assay, we analyzed the coefficient of variation (CV) among replicate UT-digital PCR quantifications of the trace 454 libraries (Table 2). The mean CV was found to be 9.0% with standard error of the mean (SEM) 1.2%, indicating that the UT-dPCR assay is precise within about 10%. In order to make a direct comparison between the reproducibility of UT-dPCR and UT-qPCR quantification, we carried out a dedicated study. A variety of 454 Libraries were assayed in replicate (six to eight replicates per library per method) by both UT-digital PCR and UT-quantitative PCR [see Additional file 2]. The real-time PCR measurements were carried out using an ideally prepared standard curve. The mean CV for UT-digital PCR assay was found to be 11.8 ± 1.5%, consistent with the results obtained by UT-digital PCR for the trace libraries and significantly lower than the CV measured for UT-quantitative PCR, 21.2 ± 2.6% (p < 0.05, t-test, Figure 3B). Because the digital assay relies on neither internal nor external standards, the CV figure of 11.8% represented in Figure 3B closely approximates the real-world accuracy of the digital assay, which is sufficient to prepare bulk emulsion PCR or bridge PCR reactions without prior titration.

Figure 3A shows that we can obtain enough library DNA from 500 pg of genomic (shotgun) or amplicon DNA to create more than 100,000 enriched (DNA-bearing) beads for sequencing. All twelve trace libraries were sequenced in a single bulk run of our GS FLX 454 DNA pyrosequencer. In total, 18 million raw bases were sequenced from the trace shotgun libraries and 38 million raw bases were sequenced from the amplicon libraries. 69.16% of the shotgun reads and 99.17% of the amplicon reads mapped back to *E. coli*. Specifically, in the case of the library made from 500 pg of *E. coli *16S amplicon, half of the resulting library was used for sequencing. 14.0 million raw bases were obtained in 55,206 reads with 99.02% of the reads mapping back to the template, indicating that almost 30 Mbp can be obtained from a library of 131,000 molecules prepared from 500 pg input material, in this case at a specific yield of 220,824 454 FLX reads (254 bp reads) per nanogram input DNA. Similarly, half of the 1 ng *E coli *amplicon library gave 10.9 million raw bases in 43,217 reads with 99.17% mapping. The 500 pg *E coli *shotgun library gave 5.7 million raw bases in 26,812 reads (69.9% mapping), while the 1 ng *E coli *shotgun library gave 6.0 million raw bases in 28,730 reads (69.9% mapping). These results indicate that better than one-fold coverage of the ~4.7 Mbp K12 genome can be obtained from less than 1 ng input DNA. Detailed information about these sequencing results is presented in Table 3.

**Table 3:** 454 FLX trace library sequence results

***Sample ID***	***Organism***	***Library Type***	***input (ng)***	***Proportion of library sequenced***	***Raw bases (Mbp)***	**Number of reads**	***Average read length (bp)***	% mapping to template/assembling*
***TS-5***	*E. coli*	Shotgun	1.0 ng	1.0	6.0	28730	210.5	69.9%
***TS-6***	*E. coli*	Shotgun	0.5 ng	1.0	5.7	26812	212.5	69.9%
***TS-11***	*E. coli*	Amplicon	1.0 ng	0.5	10.9	43217	252.5	99.2%
***TS-12***	*E. coli*	Amplicon	0.5 ng	0.5	14.0	55206	253.6	99.0%
***IgG***	*M. Musclus*	Shotgun	~1.2 ng	1.0	3.8	27712	139.8	10.1%
***K27-1***	*M. Musclus*	Shotgun	~1.2 ng	1.0	4.1	27701	147.7	35.3%
***K27-2***	*M. Musclus*	Shotgun	~1.2 ng	1.0	7.1	42829	166.0	21.0%
***pX***	*H. Sapien*	Shotgun	4.9 ng	0.21	42	244010	172.6	64.6%
***Ace***	*A. longum*	Shotgun	723 ng	0.005	67	278181	240.9	98.8%*

2,400,000 sequencing library molecules (or 0.71 pg amplifiable DNA) from an *Acetonema longum *shotgun library (prepared according to the standard library preparation method from 723 ng of genomic DNA) were sufficient for digital PCR, emulsion PCR and sequencing on the 454 FLX. From these molecules, 74% of the beads loaded gave useful 454 sequence data (4.13% 'mixed' reads and 4.28% 'dot' reads) to yield 67 Mbp in 278,181 reads on one large PTP region (one-half of the 454 FLX sequencing run). Together with 38 Mbp of shotgun data from another run, 105.6 Mbp of very high quality *Ace *shotgun data were obtained without any titration techniques, 104.3 Mbp of which assembled *de novo *under Newbler to give better than 20-fold coverage of the ~5 Mbp *Acetonema longum *genome with N50 contig size in excess of 50,000 bp. These results indicate that significant quantities of DNA pyrosequencing data can be obtained from subnanogram DNA samples without titration runs.

**Figure 4:**
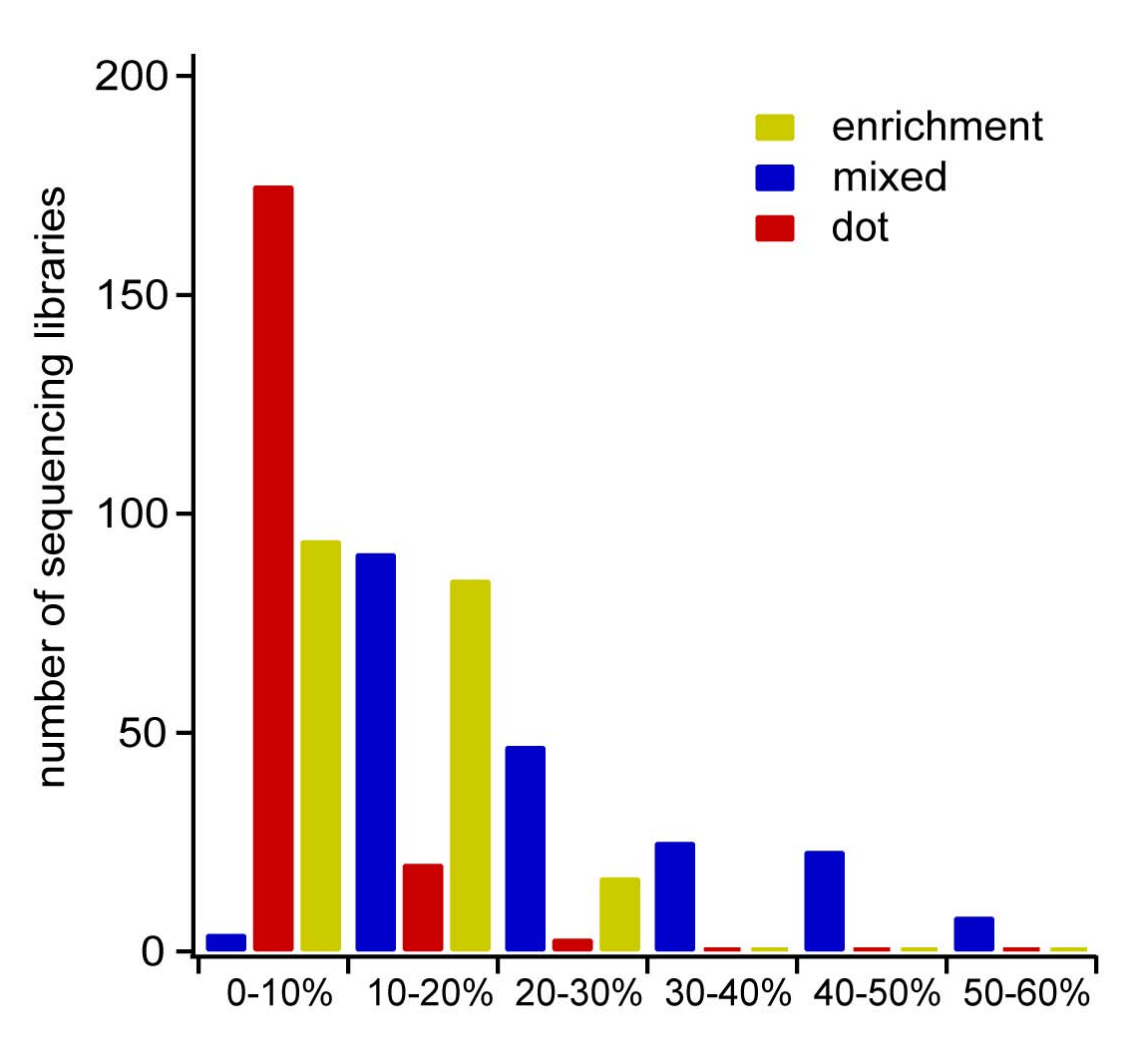
**Emulsion and sequencing metrics from 454 FLX sequencing**. 198 libraries ranging in sample type (mammalian, microbial, viral, environmental, clinical), sample quantity (0.5 ng to 5 μg), and quality (high-quality to significantly degraded) were prepared for sequencing based on UT-digital PCR quantification. Histogram of bead enrichment, 'mixed', and 'dot' percentages obtained in 454 sample preparation and sequencing when digital PCR is used for quantification. The manufacturer's recommended enrichment range is 10% to 15%, while the percentage of reads rejected as 'mixed' or 'dot' should be minimized.

We have sequenced 198 454 libraries without a single titration run using the digital PCR quantification method (selected libraries are described in Tables 2 and 3). These libraries, despite dramatic differences in source, type, molecular weight, and quality, gave acceptable emulsion PCR results as indicated by enrichment recoveries clustering below 20% within a narrow range of DNA to bead ratios, 0.08 – 0.30 DNA per bead (Figure 4). The enrichment results are validated by the 'mixed' sequencing statistic, which indicates the fraction of beads in the run that give signals indicating a likelihood that the attached DNA molecules are of nonuniform sequence.

### UT-digital PCR assay enables trace Solexa library quantification and sequencing

A similar UT-digital PCR assay was designed to quantify Solexa sequencing libraries (see methods for details). Solexa libraries were prepared from human plasma DNA or whole blood genomic DNA using starting amounts of DNA between 2 and 6 ng. The concentrations of library molecules were determined by UT-digital PCR, and diluted to 4 pM for loading onto the sequencer. We achieved a consistent cluster density between 110,000 and 150,000 clusters per tile on the Genome Analyzer II, a range deemed optimal by the manufacturer. The total number of reads obtained was 11 to 15 million per lane (Table 4). In the case of the whole blood sample, where a library was prepared from 2.1 ng DNA, in excess of 300 Mb of raw sequence data were obtained. We also made an attempt to quantify the Solexa libraries on the Agilent Bioanalyzer and NanoDrop spectrophotometers. Had we determined the dilutions based on the results from these standard techniques, we would have obtained cluster densities too high and too low by factors of two, respectively.

**Table 4:** Solexa trace library generation & sequence results

**Solexa Libraries**	**Input (ng)^a^**	DNA Library (total molecules by UT-dPCR)*/ul	**Average number of clusters generated per tile**	**Total number of reads**	**% mapping to Human reference (hg 18)**
Plasma DNA Sample 1	3.2	1.07 × 10^11^	115998	11599833	51.5%
Plasma DNA Sample 2	3.6	7.88 × 10^10^	114548	11454876	52.7%
Plasma DNA Sample 3	2.7	7.17 × 10^10^	118516	11851612	56.1%
Plasma DNA Sample 4	2.6	6.03 × 10^10^	150414	15041417	49.7%
Plasma DNA Sample 5	5.6	7.17 × 10^10^	119104	11910483	56.1%
Plasma DNA Sample 6	2.4	7.23 × 10^10^	120974	12097478	55.4%
Whole Blood DNA Sample	2.1	6.30 × 10^10^	151201	15120171	50.5%

^a ^Quantified by digital PCR with human specific primers on a unique locus taking 6.6 pg per cell.

## Discussion

Environmental and clinical sampling for diagnostic, forensic, and metagenomic applications often yields mere nanograms of genetic material, an amount presently considered insufficient to support next-generation library preparation. Common practice is to amplify the materials using PCR or whole genome amplification, methods which introduce bias to the overall representation of the sample on an intentional or unintentional basis. There exists a clear need for a straightforward and reliable method to bring nanogram and subnanogram samples onto the next-generation sequencing platforms.

Quantifying the sequencing libraries by mass, as recommended in the sequencing protocols, presents three major stumbling blocks that render the quantification inaccurate to the degree where the sequencing results are compromised. First, mass-based quantification requires an accurate estimate of the length of the molecules to determine the molar concentration of DNA fragments. Second, degraded and damaged molecules that cannot be amplified in the massively parallel amplification step are counted. And third, methods of measuring DNA mass lack sensitivity, and are inaccurate at or below low-nanogram quantities.

Quantitative real-time PCR, and especially digital PCR, are ideal candidate techniques for this application because of their exquisite sensitivity. Some detection chemistries for real-time PCR, such as TaqMan, have the property of counting molecules rather than measuring DNA mass, although in the real-time modality, the measurements are relative and the methods by which standards are established often tie the real-time PCR results back to mass.

Recently, Meyer *et al*. developed a SYBR Green real-time PCR assay that allows the user to estimate the number of amplifiable molecules in sequencing libraries [11]. This was the first report of PCR-based quantification of sequencing libraries, and extended the sensitivity of library quantification significantly – although to an unknown extent, since the source material used to make the Neandertal (presumably the lowest input quantity) libraries was not quantified. However, the SYBR Green assay presents several disadvantages: SYBR Green I dye is an intercalating flurochrome that gives signal in proportion to DNA mass, not molecule number; SYBR Green assays rely on external standards that limit the absolute accuracy and are not universal to all sample types; finally, intercalating fluorochromes give signal from nonspecific PCR reaction products. After this manuscript was submitted, a report from the Sanger Center describing the use of real time Taqman PCR to quantify sequencing libraries appeared [12]. While this eliminates some of the problems related to SYBR Green, it was not applied to trace libraries and suffers from the same drawbacks as all real-time assays.

In a real-time assay, the standard must have the same amplification efficiency and molecular weight distribution as the unknown library sample. This means the user must have on hand a bulk sequencing library very similar to the trace library being made and that the molecular weight distributions of both the standard and the new library be known – often an impractical requirement for low-concentration shotgun libraries. Furthermore, this standard library must be of extremely high quality if mass-based quantification is to be used to calibrate the assay for amplifiable molecules. If not, the concentration of all the unknown samples will be overestimated, and the yield of enriched beads or clusters will be poor. For this reason, Roche and Illumina recommend carrying out a four-point titration run on their sequencers to empirically determine the quantity of DNA to be used before carrying out a bulk sequencing run with a new library. In addition, Illumina recommends that the user check the library quality with traditional Sanger sequencing before its application to high-throughput sequencing.

Lastly, sequence-nonspecific detection chemistries like SYBR Green give signal from all dsDNA products generated, including primer dimers and nonspecific amplification products, which can be a severe issue in complex samples. In particular, side products can compete with specific amplification from low numbers (<1000) of template molecules, limiting the accuracy of SYBR Green quantification for dilute samples [13]. Although the presence of these side products can often be discerned by analysis of the product melting curve, opportunities to optimize the primers are limited due to the short length of the adaptor sequences and the specific nucleotide sequences required for compatibility with proprietary sequencing reagents. Sensitivity to side products gives SYBR Green a tendency toward overestimation of the sample quantity.

The characteristics of the quantification methods discussed are summarized in Table 1. The digital PCR method eliminates the issues associated with mass-based quantification and real-time PCR, as well as the requirement for titration, significantly reducing the cost of preparing a library for bulk sequencing. For example, the marginal cost of titrating a 454 library on the sequencer according to the manufacturer's protocol is $1500 – $2000, while the cost to quantify a sequencing library on the digital PCR chips is $30 – $90, depending on the number of panels dedicated to each library (typically 1 – 3 panels per library). In addition, PCR-based quantification saves time and leaves the expensive sequencing instrument free to carry out bulk sequencing runs.

## Conclusion

Our results demonstrate that significant quantities of high quality sequencing data can be obtained from nanogram quantities of genetic material with the aid of digital PCR quantification. Digital PCR quantifies the amount of DNA by counting the number of positive amplification reactions from individual DNA molecules independent of amplification efficiency, and requires no standard, calibration, or information about the molecular weight distribution of the template molecules. The extraordinary sensitivity of real-time and digital PCR eliminate quantification as a material-limiting step in the sequencing workflow, bringing greater focus to library preparation procedures as the next most limiting step in sequencing trace samples. It is natural to expect that library preparation protocols developed with the capacity to handle up to five micrograms of input are far from optimal with respect to minimizing loss from nanogram or picogram samples. A procedure optimized for trace samples with reduced reaction volumes and media quantities, possibly formatted in a microfluidic chip, has the potential to dramatically improve the recovery of library molecules, allowing preparation of sequencing libraries from quantities of sample comparable to that actually required for the sequencing run, *e.g. *close to or less than one picogram.

Digital PCR quantification is sufficiently accurate in counting amplifiable library molecules to justify elimination of titration techniques as well as the associated time and cost. The method is also hundreds of millions of times more sensitive than traditional means of library quantification, and allows the sequencing of libraries prepared from tens to hundreds of picograms of starting material, rather than the micrograms of DNA required by the manufacturers' protocols. The reduced sample requirement enables the application of next-generation sequencing technologies to minute and precious samples without the need for pre-amplification.

## Methods

### Sample generation

DNA was extracted from mid-log phase *E. coli *K12 and and *Acetonema longum *cultures using Qiagen's DNeasy Tissue & Blood kit and further purified using Qiagen's QIAquick PCR purification kit following the manufacturer's protocol. *E*.*coli *amplicons were generated from 16S rRNA PCR following standard protocols to generate a uniform 466 bp fragment. Sample pX and Solexa libraries were DNA extracted from human plasma or whole blood using Qiagen's DNA Blood Mini Kit or Machinerey-Nagel's NucleoSpin Plasma Kit according to manufacturers' protocols. Samples K27-1, K27-2, IgG consisted of purified mouse DNA from chromatin immunoprecipitation experiments. The initial *E. coli *DNA template and *Acetonema longum *sample used for 454 FLX sequencing were quantified by Nanodrop, Agilent Bioanalyzer DNA chip, and a 16S rRNA qPCR assay. The *E. coli *template was further diluted to the 0.5 – 35 ng range for samples TS 1 – 6 prior to library construction. The *E. coli *template for samples TS 7 – 12 was PCR-amplified from 3 ng of initial template. The PCR product was quantified by Nanodrop, Bioanalyzer DNA chip, and 16S rRNA qPCR, then aliquotted to the final amounts (0.5 – 35 ng) before library construction. For samples K27-1, K27-2, and IgG, the initial template DNA was quantified by Nanodrop and Aligent Bioanalyzer. Sample pX was quantified by Nanodrop, Agilent Bioanalyzer, and digital PCR with human-specific primers.

### Sequencing library preparation

454 shotgun libraries were generated according to the manufacturer's protocol with a few adjustments: trace *E*.*coli *amplicons and human sample pX were not nebulized; 0.01% Tween-20 was added to the elution buffer for each mini-elute column purification step; and libraries were eluted using 1xTE containing 0.05% Tween-20 at a volume of 30 μl. Single-stranded libraries were aliquotted for storage. Solexa libraries were generated following standard genomic DNA protocol with the following adjustments: no nebulization was performed on plasma DNA samples since they were fragmented in nature (average ~170 bp); the whole blood genomic DNA sample was sonicated to produce fragments between 100 and 400 bp; all ligated products were used for 18-cycle PCR enrichment; no gel extraction was performed; and no Sanger sequencing was used to confirm fragments of correct sequence.

### Standard creation for UT-quantitative PCR on the Statagene Mx3005

After sequencing library preparation, UT-quantitative PCR was used to range the concentration for UT-digital PCR. For use with UT-quantitative PCR, a standard library was created, quantified by UT-digital PCR and serially diluted for use as a UT-quantitative PCR standard. In order to ensure uniform amplification among various libraries, several standard samples were prepared such that in each UT-quantitative PCR the fragment length distribution and average GC content of the standard approximated those of the samples being quantified.

### UT-quantitative PCR quantification on the Statagene's Mx3005

Validated standards were diluted in ten-fold increments through the range 10^15^–10^3 ^molecules/μl. Standards were assayed in triplicate in order to obtain standard deviation/relative coefficient of variation. Each library was diluted ten-fold, and assayed with twelve replicates in order to obtain standard deviation/relative coefficient of variation. The thermal cycling parameters are listed in Table 5.

**Table 5:** Thermocycling parameters for UT-quantitative PCR and UT-digital PCR

	**Standard Adapters 454** **UT-dPCR & UT-qPCR**	**MIDs/Paired-end** **UT-qPCR**	**MIDs/Paired-end** **UT-dPCR**	**Solexa** **UT-dPCR**
**Hot Start**	95C, 3 mins	95C, 3 mins	95C, 3 mins	95C, 10 mins
**Denaturation**	94C, 30 secs	95C, 3 secs	95C, 15 secs	95C, 15 secs
**Annealing**	60C, 30 secs	65C, 30 secs	65C, 30 secs	60C, 1 min
**Extension**	72C, 45 secs	-	-	-
**Cycle**	40	40	40	40

### UT-digital PCR quantification on Fluidigm's BioMark System

454 libraries: UT-quantitative PCR was first performed on aliquotted libraries in order to estimate the dilution factor for UT-digital PCR. The libraries were diluted to roughly 100–360 molecules per μl. PCR reaction mix containing the diluted template was loaded onto Fluidigm's 12.765 Digital Array microfluidic chip. The microfluidic chip has 12 panels and each panel contains 765 chambers. The concentration of diluted template that yielded 150–360 amplified molecules per panel was chosen for technical replication. Six replicate panels on the digital chip were assayed in order to obtain absolute quantification of the initial concentration of library.

Solexa libraries: quantitative real-time PCR using human specific primers was first performed to estimate the dilution factor required for carrying out UT-digital PCR. The final dilution yielded 150–360 amplified molecules per panel.

All libraries: The following reagents were used for all UT-quantitative PCR and UT-digital PCR assays: Universal Taqman Probe Master Mix (Roche) at 1× final concentration, 200 nM forward primer, 200 nM UT probe-binding primer, 400 nM reverse primer and 350 nM UPL (Universal Probe Library) #149 (Roche). The primer and probe sequences and the thermal cycling parameters are presented in Tables 5 and 6 respectively.

**Table 6:** Primer/probe list for UT-quantitative PCR and UT-digital PCR

**Primers for Standard 454 libraries:**	
**Forward:**	5'-CCATCTCATCCCTGCGTGTC-3'
**Reverse:**	5'-CCTATCCCCTGTGTGCCTTG-3'
**UTBP-1:**	5'-GGCGGCGACCATCTCATCCCTGCGTGTC-3'
**Primers for 454 MID/Paired end libraries:**	
**Forward:**	5'-GCCTCCCTCGCGCCATCAG-3'
**Reverse:**	5'-GCCTTGCCAGCCCGCTCAG-3'
**UTBP-2:**	5'-GGCGGCGAGCCTCCCTCGCGCCATCAG-3'
**Primers for Solexa libraries:**	
**Forward:**	5'-ACACTCTTTCCCTACACGA-3'
**Reverse:**	5'-CAAGCAGAAGACGGCATA-3'
**UTBP-3:**	5'-GGCGGCGAACACTCTTTCCCTACACGA-3'
**Universal probe sequence:**	
**UPL#149**	5'-CCGCCGCT-3'

### Emulsion PCR/Bridge PCR & Sequencing

454 sequencing: Sequencing was performed according to manufacturer's protocol. No titration or Sanger sequencing was performed. The DNA to bead ratios of 0.085 – 0.300 (based on UT-digital PCR quantification) were used. These ratios resulted in acceptable enrichment sequencing results, including an incidence of 'mixed' reads clustering below 20%. 'Mixed' reads in 454 sequencing are defined as four consecutive positive nucleotide flows for a given read. Solexa sequencing: Sequencing libraries were first diluted to 10 nM according to the concentration determined by digital PCR. The average dilution factor was 10 – 20. Diluted libraries were denatured with 2 N NaOH and then diluted to a final concentration of 4 pM. The templates were loaded onto flow cells. Cluster generation was performed according to the manufacturer's instructions. Sequencing was carried out on the Genome Analyzer II. No titration or Sanger sequencing was performed.

## Abbreviations

CV: Coefficient of Variation; DNA: Deoxyribonucleic Acid; dsDNA: Double-stranded DNA; ssDNA: Single-stranded DNA; gsFLX: genome sequencer 'flex' from Roche/454; LOD: Limit of Detection; LOQ: Limit of Quantification; MDA: Multiple Displacement Amplification; MID: Multiplex Identifier; PCR: Polymerase Chain Reaction; PTP: PicoTiter Plate; SEM: Standard Error of the Mean; SGTC: Stanford Genome Technology Center; UT: Universal Template; WGA: Whole Genome Amplification.

## Competing interests

SRQ is a founder, consultant, and equity holder of Fluidigm. In addition, Stanford University may file a patent application on the results presented here.

## Authors' contributions

RAW and HCF acquired the data. All of us contributed to the experimental design, data analysis/interpretation, and drafting of the manuscript. All of us read and approved the final manuscript.

## Supplementary Material

Additional file 1 **dPCR analysis of mock library control. **The figure shows the absence of digital counts from a mock sequencing library preparation (454). False-color image of 12.765 digital array at assay endpoint. Each grid point corresponds to a nanoliter-scale PCR reaction, with yellow squares revealing amplification due to the presence of at least one sequencing library template molecule. The panels show dilution series (indicated) of a library preparation carried out as usual but for omission of sample DNA. The *Ace *sample (described in Table 2 of the main text) is used here as a positive control. Click here for file

Additional file 2 **replicate quantification of 12 test libraries by UT-dPCR and UT-qPCR.** The table shows data from the replicate quantification of 12 test libraries by UT-dPCR and UT-qPCR. Click here for file
