# Correction: Digital PCR provides sensitive and absolute calibration for high throughput sequencing

**DOI:** 10.1186/1471-2164-10-541

**Published:** 2009-11-19

**Authors:** Richard A White, Paul C Blainey, H Christina Fan, Stephen R Quake

**Affiliations:** 1 Department of Bioengineering at Stanford University and Howard Hughes Medical Institute, Stanford, California 94305, USA

## Correction

After this article [1] appeared online, an error was called to our attention. The "universal probe" sequence UPL #149 in Table 6 appears with the 5' and 3' ends reversed. The correct sequence of this locked nucleic acid (LNA) probe is 5'-TCGCCGCC-3'. This typographical error does not affect any of the conclusions drawn in the article.
