# Supplementary figures and images for: Digital PCR provides sensitive and absolute calibration for high throughput sequencing
Source: BMC Genomics. 2009 Mar 19;10:116. doi: 10.1186/1471-2164-10-116 (PMC2667538; doi:10.1186/1471-2164-10-116)

UT digital PCR on Fluidigm 12.765 digital Array

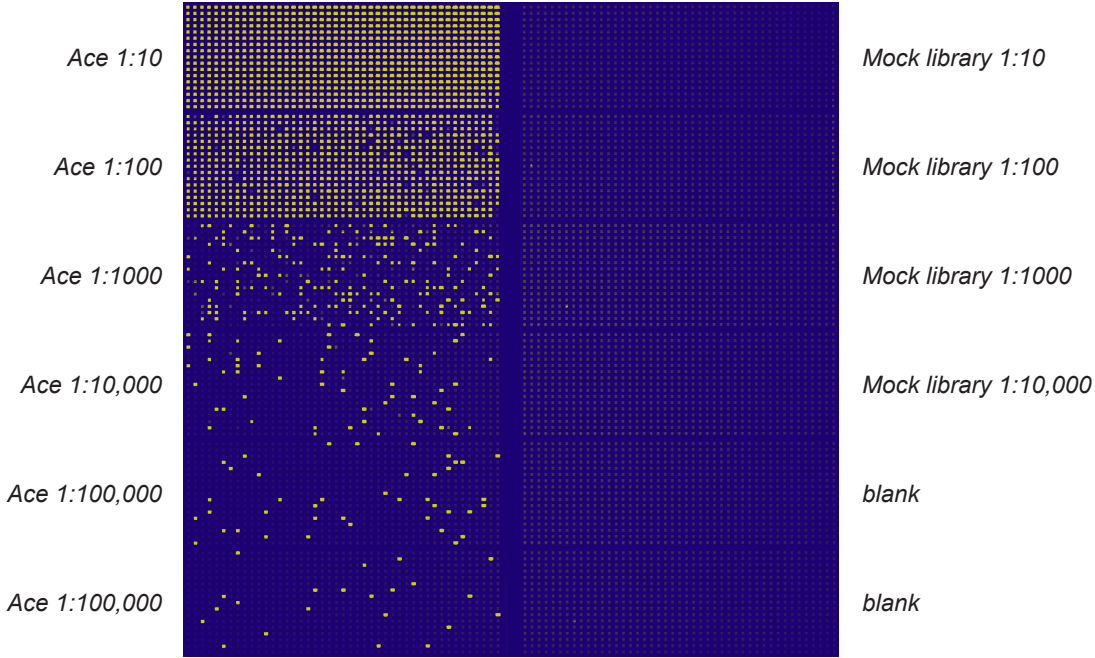

Supplement: Additional file 1 — dPCR analysis of mock library control. The figure shows the absence of digital counts from a mock sequencing library preparation (454). False-color image of 12.765 digital array at assay endpoint. Each grid point corresponds to a nanoliter-scale PCR reaction, with yellow squares revealing amplification due to the presence of at least one sequencing library template molecule. The panels show dilution series (indicated) of a library preparation carried out as usual but for omission of sample DNA. The Ace sample (described in Table 2 of the main text) is used here as a positive control. [file 1471-2164-10-116-S1.pdf]
