# Supplementary material for: Digital PCR provides sensitive and absolute calibration for high throughput sequencing
Source: BMC Genomics. 2009 Mar 19;10:116. doi: 10.1186/1471-2164-10-116 (PMC2667538; doi:10.1186/1471-2164-10-116)
Supplement: Additional file 2 — replicate quantification of 12 test libraries by UT-dPCR and UT-qPCR. The table shows data from the replicate quantification of 12 test libraries by UT-dPCR and UT-qPCR. [file 1471-2164-10-116-S2.pdf]

Sample ID	***Library Type***	***input (ug)***	***Mean Library fragment size (bp)***	***Input (total molecules by mass)***	***ssDNA library (total molecules by UT-dPCR)***	***dPCR replicate CV***	***ssDNA library (total molecules by UT-qPCR)***	***qPCR replicate CV***
Test Library #1	Shotgun	11.3	350	5.92^{13}	6.08^{8}	12.76%	4.90^{4}	12.16%
Test Library #2	Shotgun	0.9	350	4.89^{12}	1.05^{7}	17.90%	1.94^{6}	21.99%
Test Library #3 (ACE)	Shotgun	0.7	550	2.41^{12}	1.62^{6}	13.17%	6.79^{5}	19.03%
Test Library #3	Amplicon	4.1	400	1.89^{13}	5.54^{8}	5.86%	4.04^{5}	18.19%
Test Library #4	Amplicon	7.9	400	3.62^{13}	8.82^{8}	14.19%	6.33^{7}	32.20%
Test Library #5	Amplicon	7.2	400	3.28^{13}	4.60^{8}	15.43%	4.50^{6}	37.70%
Test Library #6*	Amplicon	5.0	35	2.62^{14}	3.16^{10}	12.41%	3.18^{9}	28.42%
Test Library #7*	Amplicon	5.0	35	2.62^{14}	1.92^{10}	4.38%	9.92^{9}	29.89%
Test Library #8	Shotgun	2.4	350	1.24^{13}	3.10^{7}	18.00%	9.42^{5}	12.89%
Test Library #9	Shotgun	3.9	350	2.06^{13}	5.16^{7}	15.80%	1.23^{7}	7.86%
Test Library #10*	Amplicon	5.0	35	2.62^{14}	1.63^{7}	7.70%	1.01^{5}	15.83%
Test Library #11*	Amplicon	5.0	35	2.62^{14}	3.19^{7}	3.94%	9.11^{7}	18.00%
					**mean CV**	**11.80%**	**mean CV**	**21.18%**
*Libraries prepared/sequenced at SGTC					**CV SEM**	**1.46%**	**CV SEM**	**2.62%**
